# The Relevance of Obesity for Activities of Daily Living in Geriatric Rehabilitation Patients

**DOI:** 10.3390/nu13072292

**Published:** 2021-07-01

**Authors:** Julia Wojzischke, Jürgen M. Bauer, Andreas Hein, Rebecca Diekmann

**Affiliations:** 1 Department of Health Services Research, Assistance Systems and Medical Device Technology, Carl von Ossietzky University Oldenburg, 26129 Oldenburg, Germany; julia.wojzischke@uol.de (J.W.); andreas.hein@uol.de (A.H.); 2 Center for Geriatric Medicine and Network Aging Research, Heidelberg University, 69126 Heidelberg, Germany; juergen.bauer@bethanien-heidelberg.de

**Keywords:** obesity, body mass index, waist circumference, fat mass, activities of daily living, Barthel index, geriatric rehabilitation, long-term

## Abstract

The obesity pandemic has reached old age but the effect of obesity on functional recovery in geriatric rehabilitation patients has not been investigated to date. In this prospective cohort study, patients admitted into geriatric rehabilitation were consecutively included between September 2015 and September 2016, aged ≥70 years. Individual activities of daily living were documented by the Barthel index (BI, 0–100 points). Obesity was assessed by the measurement of body mass index (BMI, kg/m²), waist circumference (WC, cm) and percentage of body fat mass (%FM) based on triceps’ skinfold thickness at admission (t1), discharge (t2) and six months after discharge (t3). A total of 122 patients were included in the analysis. Prevalence of obesity according to BMI, WC and %FM was 33.6%, 83.6% and 71.3% respectively. Patients with a high WC and patients with a high BMI had lower BI values at t1, t2, t3 and the improvement in BI (t1–t2, t2–t3) was lower than in those with low WC and low BMI, but without statistical significance. In multiple regression analysis, BMI, WC and %FM were not associated with BI at t3 and improvement of BI (t2–t3). Obesity was highly prevalent in geriatric rehabilitation patients, but it was not associated with BI during the 6-month follow-up.

## 1. Introduction

It has been shown that nutritional status is associated with functional outcomes in geriatric rehabilitation patients [1,2] and malnutrition was associated with a decreased improvement in activities of daily living (ADL) over the course of geriatric rehabilitation [2]. However, the relevance of obesity for functional outcomes has only rarely been investigated in this setting, although this seems particularly relevant in view of the obesity pandemic in old age. According to the published scientific literature, mild obesity appears to be a protective factor for functionality, whereas excessive obesity should be considered as a risk factor in this regard [3].

At the moment, age-specific cut-off points for the diagnosis of obesity are still under discussion [4]. According to the World Health Organization (WHO), obesity is defined as an abnormal and excessive fat accumulation, accompanied by an increased risk for negative health outcomes [5]. For practical reasons, obesity diagnosis has often been limited to the measurement of BMI. However, BMI does not take into account age-related changes in body composition and height. Alternative approaches include an elevated percentage of body fat (%FM) of ≥35% in women (w) and ≥25% in men (m) and an increased waist circumference (WC) of ≥88 cm (w) and ≥102 cm (m) [4]. To date, studies on geriatric rehabilitation patients on the relationship between obesity and ADL focused on BMI only and included no follow-up data. Furthermore, the respective studies did not explore the relevance of obesity but used the BMI more as a co-variable. 

Therefore, the aim of this study was to investigate the association between ADL and obesity parameters—BMI, WC and %FM according to triceps skinfold thickness (TST)—during geriatric rehabilitation and at six-months follow-up.

## 2. Materials and Methods

### 2.1. Subjects and Study Design

Patients aged 70 years and older, admitted between September 2015 and September 2016 to the geriatric rehabilitation ward of the rehabilitation center in Oldenburg, Germany, were enrolled in this prospective cohort study. Exclusion criteria were the inability to understand the content and the course of the study. Geriatric rehabilitation in Germany is characterized by inpatient rehabilitation with a structured rehabilitation program to regain independence and functionality in older persons with reduced functional conditions due to preceded acute medical events (e.g., fractures, joint replacements, cardiac infarction, surgical interventions) or chronic diseases (e.g., heart failure, Parkinson`s disease, frailty). Geriatric rehabilitation is interdisciplinary and includes medical care, nursing care, physiotherapy, occupational therapy, neuropsychology and social services. Nutritional therapy and speech therapy are applied if necessary. The length of stay in geriatric rehabilitation depends on the patient’s health insurance and the rehabilitation progress. It ranges between 18 and 28 days. The study protocol was approved by the medical ethics committee of Hannover University (study number 6814), and informed consent was obtained from all patients before enrolment into the study.

Baseline obesity status, ADL and the patient’s general characteristics were collected within the first three days of geriatric rehabilitation (t1). Follow-up data of ADL were obtained within the last three days of geriatric rehabilitation (t2) and six months after discharge from geriatric rehabilitation via telephone interview (t3). 

### 2.2. Obesity

Obesity was measured by BMI, WC and %FM according to TST measurement. BMI (kg/m²) was calculated by using body weight and body height in the following equation: body weight (kg) divided by the square sum of body height (m). Body height (cm) and body weight (kg) were measured to the nearest of 1.0 cm and 0.1 kg, respectively, with a stadiometer, including an integrated body scale, the Seca 285 (Seca, Hamburg, Germany). According to BMI, patients were allocated into two groups: high BMI (≥30 kg/m²), normal and low BMI (≤29 kg/m²). WC was measured to the nearest 0.1 cm while standing with a tape measure perpendicular to the body, with the axis in the middle between the lowest costal arch and the upper end of the pelvic bone. WC groups were determined by high WC (≥88 cm females and ≥102 cm males) and normal WC (≤87 cm females and ≤87 cm males). The total body fat mass (kg) and the %FM were estimated based on TST measurement. TST (mm) was measured at the right side of the body on the relaxed arm in the middle between the acromion tip and the olecranon process to the nearest 0.2 mm using a skinfold caliper (GPM, Zurich, Switzerland). The %FM was calculated according to the formula of Siri 1956 [6]:

Density [kg/m²] male = 1.1041 − 0.0662 × log TST [mm](1)

Density [kg/m²] female = 1.1160 − 0.0762 × log TST [mm](2)

Total body fat mass [kg] = (4.95/density − 4.50) × body weight [kg](3)

%FM [%] = total body fat mass [kg] / body weight [kg] × 100 [%](4)

Fat mass groups were defined as high %FM (≥35% females and ≥25% males) and normal %FM (≤34% females and ≤24% males). The BMI, WC and TST measurements were carried out by the study staff. 

### 2.3. Activities of Daily Living

The rehabilitation outcome was based on ADL according to the BI. The BI is a validated, and commonly used, 10-item scale with a value between 0 and 100 points. The ability of the following ADLs is assessed by the BI: feeding, bathing, grooming, dressing, toileting, chair transfer, ambulation, stair climbing, bowel control and bladder control. BI at t1 and t2 was assessed by the nursing staff and obtained from the electronic patient record. BI at t3 was based on a patient’s self-assessment, obtained by an interview-administered questionnaire via telephone call [7].

### 2.4. General Characteristics

A patient’s general characteristics were obtained at t1 by an interview-administered questionnaire including questions on sex, age, and stay before rehabilitation. Information on the leading and secondary diagnoses, the Mini Mental Status Examination (MMSE, 0–30 points) [8] and the Geriatric Depression Scale (GDS, 0–15 points) [9] was obtained from the electronic patient record. MMSE and GDS were recorded by a neuropsychologist. The Charlson Comorbidity Index (0–35 points) [10] was calculated based on the recorded diagnoses. Malnutrition status was assessed by the Mini Nutritional Assessment Short Form (MNA-SF, 0–14 points) [11]. Handgrip strength was measured three times for both hands with a Jamar hydraulic hand dynamometer (J.A. Preston Corporation, Clifton, USA), and the maximum value was recorded. 

### 2.5. Statistics

Statistical analysis was performed with IBM SPSS Statistics (version 25.0). All parameters were tested for normal distribution by skewness. Normal distributed variables are represented in mean ± standard deviation (SD); not normally distributed variables are represented in median (25–75. percentiles). Due to the explorative character of the study no sample size calculation was performed beforehand. The results should be interpreted as explorative and should be confirmed in a confirmatory study. Testing for group difference was calculated by *t*-test for metric, normally distributed variables, Mann–Whitney-U-test for ordinal and not normally distributed variables, and X²-test for nominal variables. The relationship between obesity and ADL according to BI was evaluated using a multiple linear regression analysis. 

## 3. Results

In total, 150 geriatric rehabilitation patients were initially included in the present study. A total of 28 patients had to be excluded as they dropped out between t1 and t2 due to the following reasons: readmission to hospital (*n* = 14), premature termination of geriatric rehabilitation (*n* = 6), burden due to study participation (*n* = 3), decreased general condition (*n* = 3), anxiety because of stumbling during the assessment (*n* = 1), patient could not be reached (*n* = 1). Between t2 and t3, a total of 13 patients dropped out for the following reasons: due to death (*n* = 4), contact was not possible (*n* = 5), burden due to study participation (*n* = 2), decreased general condition (*n* = 1), rejection of telephone interview without any given reason (*n* = 1). 

### 3.1. General Characteristics

The 122 patients who completed geriatric rehabilitation (t2) were included in the following analysis. Multiple linear regression analysis was performed in 109 patients as 13 patients did not complete the six-month follow-up (t3). Mean age (±SD) was 81.5 ± 5.6 years and 69.7% (*n* = 85) were female. Of the total, 49.2% (*n* = 60) had an orthopedic diagnosis, followed by 19.7% (*n* = 24) with a cardiologic diagnosis, and 13.1% (*n* = 16) with a neurologic diagnosis, and 18% (*n* = 22) had another diagnosis. The majority of the patients were either malnourished (31.1%, *n* = 38) or at risk of malnutrition (59.8%, *n* = 73), according to MNA-SF. An overview of the characteristics of study participants according to groups of BMI, WC and %FM is shown in Table 1. 

### 3.2. Obesity

Table 2 summarizes the body composition parameter of participants. Mean BMI ± SD was 28.2 ± 4.6, mean WC was 102.9 ±12.1 cm and %FM was 36.3 ± 6.3. A high BMI was prevalent in 33.6% (*n* = 41). Prevalence of high WC and high %FM were 83.6% (*n* = 102) and 71.3% (*n* = 87), respectively. The patients of the BMI, WC, and %FM groups did not differ significantly from each other in general characteristics in a test for group difference, except for handgrip strength. Women with a high BMI showed significantly lower handgrip strength than women with a normal or low BMI (14.2 ± 7.2 vs. 14.6 ± 4.5, *p* = 0.01). Mean (± SD) length of stay was 21.3 ± 3.8 days (Table 2).

### 3.3. Association of Obesity and Activities of Daily Living

BI values at all three measurement times (t1, t2, t3), as well as the mean gain in BI over the geriatric rehabilitation period, and the gain in BI over a six-month follow-up period, were higher in normal WC patients compared to patients with a high WC, without statistical significance. The same was observed for BMI except for BI at admission. The %FM groups showed heterogeneous results (Table 1). In two multiple linear regression models (Table 3 and Table 4) BMI, WC and %FM were neither significantly associated with BI at t3 nor with the change in BI between t2 and t3. Another diagnosis, rather than a cardiologic, neurologic, or orthopedic diagnosis, was significantly associated with BI at t3 (*p =* 0.042); and MMSE status was significantly associated with the change in BI between t2 and t3 (*p =* 0.001).

## 4. Discussion

Obesity in geriatric rehab-patients according to BMI was high, with a prevalence of 33.6%. The vast majority of geriatric rehabilitation patients were obese according to WC and %FM, with a prevalence 83.6% and 71.3%, respectively. However, in a multiple linear regression analysis BMI, WC and %BF were not associated with BI at a six-month follow-up and the development of BI between discharge from geriatric rehabilitation and a six-month follow-up. 

To our knowledge, there are no published studies that analyzed the association between obesity and the development of ADL beyond a follow-up of three months after the end of geriatric rehabilitation. The only study that investigated the effects three-month post-discharge, by Neumann et al. [6], focused on differences in the BI between patients with a low BMI (≤21 kg/m²) and those patients in the range of normal, overweight and obese BMI values. The results of this study indicated that a low BMI was associated with a lower BI at a three-month follow-up, but the relevance of obesity in this regard remains unclear, as the obese patients constituted a joined reference group with those who had a BMI in the normal weight and overweight range. Furthermore, the diagnosis of obesity was limited to the measurement of BMI. In the present study, we additionally used waist circumference measurement and fat mass according to triceps’ skinfold thickness to identify obesity in geriatric rehabilitation patients. Future studies should investigate the validity of different approaches to diagnose obesity in older adults.

The vast majority of studies that investigated the association between BMI and ADL in older patients at the time of discharge from rehabilitation did not find significant associations, except for one study, which reported a significant correlation between BMI and ADL based on the Functional Independence Measurement (FIM) motor scores in older Japanese rehabilitation patients [12]. However, none of these studies was conducted specifically in a geriatric rehabilitation but in other settings, such as in hip fracture rehabilitation [13], cardiac rehabilitation [14], and general rehabilitation [12,15,16]. In a cross-sectional study of patients in cardiac rehabilitation, the BMI in older heart failure patients was not associated with ADL based on the BI [17]. The findings for an association of BMI and the development of ADL during the rehabilitation period are limited to a couple of studies; yet, they target the geriatric rehabilitation population. Mean BMI values (25.6 ± 4.7 vs. 25.2 ± 5.4 kg/m²) did not differ between patients with good functional gain in FIM and patients with poor functional gain in FIM at a rehabilitation and aged care unit in Italy (mean length of stay of 25.5 ± 9.3 and 27.0 ± 10.0 days respectively) [18]. In addition, the relative functional gain according to BI did not differ significantly in groups of BMI quartiles (group 1: <21.1 kg/m²; group 2: 21.1–23.8 kg/m²; group 3: 23.9–27.3 kg/²; group 4: <27.4 kg/m²) in patients at a rehabilitation and aged-care unit in Italy [19]. However, BMI values differed significantly (24.2 ± 5.9 vs. 20.9 ± 4.7 kg/m², *p* = 0.003) between geriatric rehabilitation patients with BI improvement over the rehabilitation period and those without BI improvement (mean length of stay 32.7 ± 19.9 and 44.3 ± 41.4 days, respectively) in a study from the UK [20].

The results of the present multiple regression analysis are in line with the studies that reported no association between BMI and ADL during geriatric rehabilitation. However, those studies focused on BMI only and the analyzed study population was older patients in rehabilitation in general and not specifically mostly multimorbid geriatric patients above the age of 70 years. We observed a trend towards lower mean BI values in patients with high BMI and high WC. This preliminary result should be confirmed by a larger study. Our findings indicate that obesity is highly prevalent in geriatric rehabilitation patients in Germany (BMI 33.6%; WC 83.6%; %FM 71.3%). We are not aware of any other studies that reported prevalence rates for geriatric rehabilitation populations. However, the prevalence of 33.6% of a high BMI in the obesity range is slightly lower than the prevalence rates in obesity in community-dwelling people aged 70 years and older in the US [21] and higher than the prevalence rates in the EU [22]. 

A review of sarcopenic obesity in older adults in general proposes a link between loss of muscle mass and gain in fat mass [23]. Our results indicate no influence of obesity on ADL in the population of (former) geriatric rehab patients. This observation may be considered of high relevance as weight management is frequently discussed in geriatric rehabilitation. At present, weight loss interventions are not recommended for people aged 80 years and older because of the risk of functional decline and decreased functional recovery; weight reduction in obese older persons should be combined with physical exercise to preserve muscle [24]. The results of the current study support this recommendation for the group of geriatric rehabilitation patients. A limitation of this study is the self-assessment of BI by a telephone-administered interview at t3 compared to the objective survey by the nursing staff at t1 and t2. Malnutrition was considered as a co-variable in the multiple regression analysis, but further interactions between obesity and malnutrition are not investigated in detail here, although malnutrition and obesity do coexist. This study was carried out as an exploratory study to generate hypotheses. Confirmatory studies can now be carried out from the results. In addition, future research should focus more on body composition parameters than on BMI alone. BMI provides a crude estimation of body fat mass but cannot replace exact measurements of body fat mass with suitable methods. Muscle mass should also be considered in future studies. Body composition data should be based either on bioelectrical impedance analysis or dual energy X-ray absorptiometry. Next, large-scale prospective studies are needed to add significance to these first insights into the relevance of obesity for ADL in geriatric rehabilitation patients. 

## 5. Conclusions

A high percentage of geriatric rehabilitation patients are obese. A more precise measurement of body composition than BMI will constitute an important issue when aiming for optimized nutritional recommendations. Obesity was not associated with poorer physical function. Whether obesity might even be regarded as a preventive factor (“obesity paradox”), as in other cohorts, has yet to be clarified.

## Figures and Tables

**Table 1:** Characteristics of geriatric rehabilitation patients according to groups of body mass index, waist circumference and percentage body fat mass at t1.

Variable	Total Group	High BMI	Normal/low BMI	High WC	Normal WC	High %FM	Normal %FM
	(*n* = 122)	(*n* = 41)	(*n* = 81)	(*n* = 102)	(*n* = 20)	(*n*= 87)	(*n* = 32)
Age (years)	81.5 ± 5.6	79.9 ± 5.2	82.4 ± 5.7	81.0 ± 5.5	84.4 ± 5.5	81.0 ± 5.3	82.9 ± 6.2
Leading diagnosis							
Orthopedic Cardiologic Neurologic Others	60 (49.2%) 24 (19.7%) 16 (13.1%) 22 (18.0%)	20 (48.8%) 6 (14.6%) 6 (14.6%) 9 (22.0%)	40 (49.4%) 18 (22.2%) 10 (12.3%) 13 (16.0%)	52 (51.0%) 20 (19.6%) 13 (12.7%) 17 (16.7%)	8 (40.0%) 4 (20.0%) 3 (15.0%) 5 (25.0%)	36 (41.4%) 19 (21.8%) 14 (16.1%) 18 (20.7%)	22 (68.8) 5 (15.6) 2 (6.3) 3 (9.4)
Admission from							
Hospital Short term care Home	28 (23.0%) 30 (24.6%) 64 (52.5%)	10 (24.4%) 8 (19.5%) 23 (56.1%)	18 (22.2%) 22 (27.2%) 38 (46.9%)	27 (26.5%) 23 (22.5%) 49 (48.1%)	1 (5.0%) 7 (35.0%) 12 (60.0%)	20 (23.0%) 19 (21.8%) 45 (51.7%)	7 (21.9) 11 (34.4) 13 (40.6)
Charlson Comorbidity index (0–35 points)	2.0 ± 1.6	2.1 ± 1.5	2.0 ± 1.7	1.9 ± 1.5	2.5 ± 2.2	2.0 ± 1.6	2.0 ± 1.7
Mini Mental Status Examination (0–30 points) Normal cognition (≥25 points) Cognitive impairment (≤24 points)	27.0 (25.0–28.0) 93 (76.9%) 28 (23.1%)	27.5 (24.3–28.0) ^1^ 30 (75.0%) 10 (25.0%)	27.0 (25.0–28.5) 63 (77.8%) 18 (22.2%)	27.0 (25.0–28.0) ^2^ 78 (77.2%) 23 (22.8%)	26.0 (24.3–28.8) 15 (75.0%) 5 (25.0%)	27.0 (25.0–28.0) ^3^ 68 (79.1%) 18 (20.9%)	27.0 (23.3–29.0) 23 (71.9) 9 (28.1)
Geriatric Depression Scale (0–15 points) No Depression (≤5 points) Depression (≥6 points)	3.0 (2.0–5.0) 95 (20.2%) 24 (79.8%)	3.0 (2.0–4.3) ^4^ 31 (81.6%) 7 (18.4%)	3.0 (2.0–5.0) 64 (79.0%) 17 (21.0%)	3.0 (2.0–5.0) ^5^ 79 (79.8%) 20 (20.2%)	3.0 (2.0–4.8) 16 (80.0%) 4 (20.0%)	3.0 (2.0–4.0) ^6^ 69 (82.1%) 15 (17.9%)	3.0 (2.0–6.0) 23 (71.9) 9 (28.1)
Mini Nutritional Assessment-Short Form (0–14 points) Malnutrition (0–7 points) Risk of malnutrition (8–11 points) Normal nutritional status (12–14 points)	8.7 ± 2.3 11 (9.0%) 73 (59.8 %) 38 (31.1%)	9.1 ± 2.3 11 (26.8%) 25 (61.0%) 5 (12.2%)	8.4 ± 2.3 27 (33.3%) 48 (59.3%) 6 (7.4%)	8.8 ± 2.2 28 (27.5%) 65 (63.7%) 9 8.8(%)	7.7 ± 3.0 10 (50.0%) 8 (40.0%) 2 (10.0%)	8.8 ± 2.4 28 (32.2%) 49 (56.3%) 10 (11.5%)	8.2 ± 2.2 9 (28.1) 22 (68.8) 1 (3.1)
Handgrip strength (kg) Women Men	17.4 ± 8.3 14.8 ± 5.6 ^7^ 24.7 ± 9.0 ^14^	18.5 ± 10.8 14.2 ± 7.2 ^8^ 30.1 ± 10.6 ^15^	16.8 ± 6.7 14.6 ± 4.5 ^9^ 22.4 ± 7.4 ^16^	17.4 ± 8.8 14.2 ± 5.6 ^10^ 26..3 ± 9.8 ^13^	17.3 ± 5.5 14.0 ± 4.0 ^11^ 20.5 ± 4.9 ^11^	18.4 ± 8.6 15.0 ± 6.0 ^12^ 24.5 ± 9.2 ^17^	14.3 ± 6.8 12.4 ± 4.4 ^13^ 24.4 ± 8.9 ^18^
Barthel index (0–100 points)							
Admission Discharge Six–month follow-up	70.4 ± 10.1 83.0 ± 8.9 85.4 ± 13.1	70.6 ± 10.0 81.7 ± 8.2 83.0 ± 15.3 ^14^	70.3 ± 10.2 83.7 ± 9.2 86.6 ± 11.7 ^72^	70.1 ± 9.6 82.4 ± 8.8 84.8 ± 13.1 ^90^	71.8 ± 12.5 86.3 ± 8.7 88.2 ± 13.3 ^19^	71.1 ± 10.3 83.1 ± 9.1 86.3 ± 13.3 ^18^	67.8 ± 9.1 83.1 ± 8.7 82.7 ± 12.7 ^22^
∆ Barthel index							
Discharge-admission Six-month follow-up-discharge	12.7 ± 9.4 1.7 ± 13.6	11.1 ± 6.8 0.3 ± 16.1 ^14^	13.5 ± 10.4 2.4 ± 12.2 ^19^	12.3 ± 9.0 1.6 ± 13.8 ^20^	14.5 ± 11.5 2.1 ± 13.2 ^21^	12.0 ± 9.4 2.3 ± 14.1 ^18^	15.2 ± 9.1 −0.8 ± 12.0 ^22^

Abbreviations: BMI, body mass index; %FM, percentage body fat mass; WC, waist circumference. ^1^ *n* = 40, ^2^ *n* = 10, ^3^ *n* = 86, ^4^ *n* = 38, ^5^ *n* = 99, ^6^ *n* = 84, ^7^ *n* = 85, ^8^ *n* = 30, ^9^ *n* = 55, ^10^ *n* = 75, ^11^ *n* = 10, ^12^ *n* = 56, ^13^ *n* = 27, ^14^ *n* = 37, ^15^ *n* = 11, ^16^ *n* = 26, ^17^ *n* = 31, ^18^ *n* = 5, ^19^ *n* = 72, ^20^ *n* = 90, ^21^ *n* = 19, ^22^ *n* = 6.

**Table 2:** Obesity of geriatric rehabilitation patients at t1.

Variable	Total Group	Women	Men
	(*n* = 124)	(*n* = 87)	(*n* = 37)
Weight (kg)	75.7 ± 15.0	72.0 ± 13.4	84.5 ± 14.8
BMI (kg/m²) High BMI Normal and low BMI	28.2 ± 4.6 41 (33.6%) 81 (66.4%)	28.4 ± 4.6 30 (35.3%) 55 (64.7%)	27.8 ± 4.6 11 (29.7%) 26 (70.2%)
WC (cm) High WC Normal WC	102.9 ± 12.1 102 (83.6%) 20 (16.4%)	100.1 ± 10.9 75 (88.2%) 10 (11.8%)	109.4 ± 12.5 27 (73.0%) 10 (27.0%)
TST (mm)	19.7 (14.7–25.6) **^1^**	21.3 (16.1–25.8) ^2^	15.2 (11.3–21.3) ^3^
FM (kg)	27.7 ± 8.3 **^1^**	27.4 ± 7.9 ^2^	28.4 ± 9.3 ^3^
%FM High %FM Normal %FM	36.3 ± 6.3 **^1^** 87.0 (71.3%) 32.0 (26.2%)	37.4 ± 4.9 ^2^ 56 (67.5%) 27 (32.5%)	33.7 ± 8.2 ^3^ 31 (86.1%) 5 (13.9%)

Abbreviations: BMI, body mass index; WC, waist circumference; FM, body fat mass; %FM, percentage body fat mass; TST, triceps skinfold thickness. ^1^ *n* =119, ^2^ *n* = 83, ^3^ *n* = 36.

**Table 3:** Multiple regression analyses considering Barthel index at t3 as dependent variable (*n* = 109).

Independent Variables	Unstandardized Coefficient				*p*-Value
	B	SE	95% CI of β		
BMI	−0.497	0.603	−1.695	0.700	0.411
WC	−0.151	0.216	−0.579	0.278	0.487
%FM	−0.125	0.266	−0.652	0.403	0.640
Age	4.014	4.330	−4.586	12.614	0.356
Sex ^1^	−0.367	3.616	−7.548	6.814	0.919
Cardiologic diagnosis ^2^	−4.813	4.353	−13.458	3.833	0.272
Neurologic diagnosis ^2^	3.535	3.799	−4.010	11.080	0.355
Other diagnosis ^2^	0.268	0.130	0.009	0.526	0.042 *
Barthel Index at admission	0.415	0.479	−0.535	1.366	0.388
MMSE	−1.766	0.892	−3.537	0.005	0.051
Charlson Comorbidity Index	0.000	0.631	−1.253	1.253	1.000
MNA-SF	0.244	0.196	−0.146	0.633	0.217
Handgrip strength	0.218	0.258	−0.295	0.731	0.401
Constant	84.562	32.803	19.412	149.711	0.012 *

R²: 0.242; Abbreviations: BMI, body mass index; %FM, percentage body fat mass; MMSE, Mini Mental Status Examination; MNA-SF, Mini Nutritional Assessment Short Form. * *p* < 0.05, ^1^ reference category women, ^2^ reference category orthopedic diagnosis.

**Table 4:** Multiple regression analyses considering Δ Barthel Index (t3 – t2) as dependent variable (*n* = 109).

Independent Variables	Unstandardized Coefficient				*p*-Value
	B	SE	95% CI of β		
BMI	−0.519	0.635	−1.780	0.743	0.416
WC	−0.078	0.227	−0.529	0.373	0.732
%FM	−0.221	0.280	−0.777	0.334	0.431
Age	0.923	4.561	−8.137	9.982	0.840
Sex ^1^	0.965	3.809	−6.600	8.529	0.801
Cardiologic diagnosis ^2^	−2.965	4.585	−12.072	6.142	0.520
Neurologic diagnosis ^2^	3.485	4.002	−4.463	11.433	0.386
Other diagnosis ^2^	−0.145	0.137	−0.417	0.127	0.294
Barthel Index at admission	0.199	0.504	−0.802	1.201	0.694
MMSE	−3.305	0.939	−5.171	−1.440	0.001 *
Charlson Comorbidity Index	−0.845	0.665	−2.165	0.475	0.207
MNA−SF	0.188	0.207	−0.222	0.598	0.365
Handgrip strength	0.282	0.272	−0.258	0.823	0.302
Constant	46.689	34.554	−21.938	115.317	0.180

R²: 0.207; Abbreviations: BMI, body mass index; %FM, percentage body fat mass; MMSE, Mini Mental Status Examination; MNA-SF, Mini Nutritional Assessment Short Form. * *p* < 0.05, ^1^ Reference category women, ^2^ Reference category orthopedic diagnosis.
